# Recent Advances in *Toxoplasma gondii* Infection and Toxoplasmosis

**DOI:** 10.3390/tropicalmed9070160

**Published:** 2024-07-18

**Authors:** Vinícius Longo Ribeiro Vilela, Thais Ferreira Feitosa

**Affiliations:** Laboratory of Veterinary Parasitology, Department of Veterinary Medicine, Federal Institute of Paraíba—IFPB, Sousa 58805-345, Brazil

Toxoplasmosis, caused by the protozoan *Toxoplasma gondii*, affects nearly all warm-blooded animals, including humans, domestic animals, and both terrestrial and marine wildlife. Susceptible hosts can contract *T. gondii* primarily through transplacental transmission, consumption of animal tissues with infective cysts, and ingestion of water or food contaminated with cat feces containing sporulated oocysts [1].

*Toxoplasma gondii* has a complex, facultatively heteroxenous life cycle involving multiple developmental stages and a variety of hosts and environments. In humans, toxoplasmosis is typically subclinical in immunocompetent adults, but in immunosuppressed individuals, it can lead to encephalitis and retinochoroiditis. In pregnant women, the parasite can be transmitted to the fetus, potentially resulting in miscarriage or causing chorioretinitis, intracranial calcifications, and hydrocephalus in the developing fetus [2,3].

In animals, *T. gondii* can cause miscarriage, fetal mummification, stillbirth, and congenital disease, leading to economic losses and higher production costs. It can also be subclinical in immunocompetent animals [4]. This trait complicates diagnosis and poses a risk to human health by allowing chronically infected animals to persist and facilitating the slaughter of infected animals for human consumption [5].

The consumption of raw or undercooked meat and raw milk remains a significant source of *T. gondii* infection in humans [6]. Understanding the spread of this protozoan through seroprevalence surveys is essential for assessing public health risks and implementing effective control measures. Furthermore, the importance of describing the genetic variability of *T. gondii* through genotypic characterization studies cannot be overstated. Such studies are crucial for comprehending the pathogen’s pathogenicity and virulence, ultimately informing targeted intervention strategies.

Recent developments in the field have highlighted the need for comprehensive genetic profiling of *T. gondii*. By establishing relationships between genotype and clinical manifestations, researchers can uncover associations related to the species’ biological potential, virulence, infectivity, and resistance to drugs and vaccines. This genetic knowledge is pivotal for improving diagnostic accuracy and developing more effective therapeutic and preventive measures.

Despite significant advancements, critical gaps remain in our understanding of the genetic diversity of *T. gondii* and its implications for human and animal health. The current Special Issue has made strides in addressing these gaps by featuring studies that focus on seroprevalence, geographical distribution, and clinical cases, thereby providing valuable insights into the epidemiology and risk factors associated with *T. gondii* infection.

Although these contributions have significantly advanced our understanding of the epidemiology and seroprevalence of *T. gondii*, there is still a need for more research focused on its genetic aspects. Future research should prioritize several key areas. First, large-scale, longitudinal studies that monitor the genetic evolution of *T. gondii* in various hosts and environments would enhance our understanding of how genetic variations influence transmission dynamics and disease severity. Additionally, standardized protocols for genotypic characterization should be developed to ensure consistency and comparability across studies.

Another critical area for future investigation is the exploration of host–pathogen interactions at the molecular level. By elucidating the mechanisms by which *T. gondii* evades the host immune response and establishes chronic infections, researchers can identify new therapeutic targets. Furthermore, studies examining the impact of environmental factors on the genetic diversity of *T. gondii* will be essential for predicting and mitigating future outbreaks.

While significant progress has been made in understanding the epidemiology and seroprevalence of *T. gondii*, there is still much to be learned about its genetic diversity and pathogenicity. The recent advancements presented in this Special Issue provide a solid foundation for future research, which should aim to fill existing knowledge gaps and ultimately improve public health outcomes. By continuing to explore the genetic and molecular landscape of this pathogen, we can develop more effective strategies to combat toxoplasmosis and reduce its global burden.

## References

[1] Almeria S., Dubey J.P. (2021). Foodborne transmission of *Toxoplasma gondii* infection in the last decade. An overview. Res. Vet. Sci..

[2] Khan K., Khan W. (2018). Congenital toxoplasmosis: An overview of the neurological and ocular manifestations. Parasitol. Int..

[3] Bartholo B.B.G.R., Monteiro D.L.M., Rodrigues N.C.P., Trajano A.J.B., Jesus N.R., Cardoso F.F.O., Souza F.M., Werner H., Araujo Júnior E. (2020). Treatment of acute toxoplasmosis in pregnancy: Influence in the mother-to-child transmission. J. Obstet. Gynaecol. Can..

[4] Vilela V.L.R., Feitosa T.F., Simões S.V.D., Mota R.A., Katzer F., Bartley P.M. (2023). An abortion storm in a goat farm in the Northeast Region of Brazil was caused by the atypical *Toxoplasma gondii* genotype #13. Curr. Res. Parasitol. Vector Borne Dis..

[5] Feitosa T.F., Vilela V.L.R., Batista S.P., Silva S.S., Mota R.A., Katzer F., Bartley P.M. (2023). Genetic diversity of *Toxoplasma gondii* in goats and sheep from the Northeast Region of Brazil destined for human consumption. Curr. Res. Parasitol. Vector Borne Dis..

[6] Belluco S., Simonato G., Mancin M., Pietrobelli M., Ricci A. (2018). *Toxoplasma gondii* infection and food consumption: A systematic review and meta-analysis of case-controlled studies. Crit. Rev. Food Sci. Nutr..

